# The duration of protection against clinical malaria provided by the combination of seasonal RTS,S/AS01_E_ vaccination and seasonal malaria chemoprevention versus either intervention given alone

**DOI:** 10.1186/s12916-022-02536-5

**Published:** 2022-10-07

**Authors:** Matthew Cairns, Amadou Barry, Issaka Zongo, Issaka Sagara, Serge R. Yerbanga, Modibo Diarra, Charles Zoungrana, Djibrilla Issiaka, Abdoul Aziz Sienou, Amadou Tapily, Koualy Sanogo, Mahamadou Kaya, Seydou Traore, Kalifa Diarra, Hama Yalcouye, Youssoufa Sidibe, Alassane Haro, Ismaila Thera, Paul Snell, Jane Grant, Halidou Tinto, Paul Milligan, Daniel Chandramohan, Brian Greenwood, Alassane Dicko, Jean Bosco Ouedraogo

**Affiliations:** 1 International Statistics and Epidemiology Group, Department of Infectious Disease Epidemiology, London School of Hygiene and Tropical Medicine, London, UK; 2 Malaria Research and Training Centre, Bamako, Mali; 3 Institut de Recherche en Sciences de la Santé, Bobo Dioulasso, Burkina Faso; 4 Faculty of Epidemiology and Population Health, London School of Hygiene and Tropical Medicine, London, UK; 5 Faculty of Infectious and Tropical Diseases, London School of Hygiene and Tropical Medicine, London, UK

**Keywords:** Malaria, *Plasmodium falciparum*, Seasonal malaria chemoprevention, Malaria vaccination, RTS,S/AS01_E_

## Abstract

**Background:**

A recent trial of 5920 children in Burkina Faso and Mali showed that the combination of seasonal vaccination with the RTS,S/AS01_E_ malaria vaccine (primary series and two seasonal boosters) and seasonal malaria chemoprevention (four monthly cycles per year) was markedly more effective than either intervention given alone in preventing clinical malaria, severe malaria, and deaths from malaria.

**Methods:**

In order to help optimise the timing of these two interventions, trial data were reanalysed to estimate the duration of protection against clinical malaria provided by RTS,S/AS01_E_ when deployed seasonally, by comparing the group who received the combination of SMC and RTS,S/AS01_E_ with the group who received SMC alone. The duration of protection from SMC was also estimated comparing the combined intervention group with the group who received RTS,S/AS01_E_ alone. Three methods were used: Piecewise Cox regression, Flexible parametric survival models and Smoothed Schoenfeld residuals from Cox models, stratifying on the study area and using robust standard errors to control for within-child clustering of multiple episodes.

**Results:**

The overall protective efficacy from RTS,S/AS01_E_ over 6 months was at least 60% following the primary series and the two seasonal booster doses and remained at a high level over the full malaria transmission season. Beyond 6 months, protective efficacy appeared to wane more rapidly, but the uncertainty around the estimates increases due to the lower number of cases during this period (coinciding with the onset of the dry season). Protection from SMC exceeded 90% in the first 2–3 weeks post-administration after several cycles, but was not 100%, even immediately post-administration. Efficacy begins to decline from approximately day 21 and then declines more sharply after day 28, indicating the importance of preserving the delivery interval for SMC cycles at a maximum of four weeks.

**Conclusions:**

The efficacy of both interventions was highest immediately post-administration. Understanding differences between these interventions in their peak efficacy and how rapidly efficacy declines over time will help to optimise the scheduling of SMC, malaria vaccination and the combination in areas of seasonal transmission with differing epidemiology, and using different vaccine delivery systems.

**Trial registration:**

The RTS,S-SMC trial in which these data were collected was registered at clinicaltrials.gov: NCT03143218

**Supplementary Information:**

The online version contains supplementary material available at 10.1186/s12916-022-02536-5.

## Background

Seasonal malaria chemoprevention (SMC) is now deployed at scale in West Africa, with approximately 33.5 million children receiving treatments in 2020 [1]. Sulfadoxine-pyrimethamine plus amodiaquine (SP-AQ), the drug combination used for SMC, remains highly effective at preventing malaria morbidity and mortality in the Sahel and sub-Sahel [2]. When SMC was introduced in Burkina Faso and the Gambia, the burden of uncomplicated malaria cases and malaria deaths in the context of SMC was estimated to be approximately 45–55% lower than the burden without SMC [3]. However, the burden of malaria in young children remains high in several countries where high SMC coverage has been achieved [1, 3].

A recent trial conducted in Burkina Faso and Mali showed that the combination of SMC and seasonal vaccination with the RTS,S/AS01_E_ malaria vaccine was markedly more effective than either intervention given alone in preventing clinical malaria, severe malaria requiring admission to hospital, and deaths from malaria [4]. Relative to SMC, the current standard of care, the protective efficacy of the Combined intervention against these outcomes was 62.8% (95% CI: 58.4, 66.8), 70.5% (95% CI: 41.9, 85.0) and 72.9% (95% CI: 2.9, 92.4), respectively. It is thought that the RTS,S/AS01_E_ vaccine may add to protection in children receiving SMC because its longer mode of action provides protection outside the months covered by SMC, when children are still highly exposed to malaria transmission, and because it adds to the incomplete protection from SMC during the months when SMC is administered. It was hypothesised that seasonal administration of the vaccine, with the third dose of the primary series (and single-dose annual booster(s)) administered one month before SMC starts, might optimise this impact by aligning the period of peak protection from the vaccine with the period of highest malaria risk [5].

On 6 October 2021, the World Health Organisation (WHO) recommended the deployment of the RTS,S/AS01_E_ malaria vaccine in areas with persisting high malaria transmission, including the possibility of seasonal administration in areas with seasonal transmission [6, 7]. With the potential for the combination of SMC and vaccination with RTS,S/AS01_E_ to be deployed at scale to protect children under 5 years of age, it is important to understand in more detail the extent and duration of additional protection provided when the interventions are given in combination, compared to either intervention given alone. This has implications for how these two interventions might best be timed in relation to the period of peak risk, and in relation to each other. In turn, this might have implications for which delivery systems might be most appropriate in different locations, balancing the potentially competing priorities of ideal timing, achieving high coverage, and cost-effectiveness and sustainability of delivery.

To help inform decisions that might be taken by policy makers and programme managers on how to combine SMC and seasonal malaria vaccination for optimal impact in areas with differing patterns of seasonal transmission, and to help inform mathematical modelling analyses that might support these decisions, we undertook a secondary analysis of data from the seasonal malaria vaccination trial. The purpose of these analyses was to estimate (1) how the efficacy of the RTS,S/AS01_E_ malaria vaccine changes over time, when administered seasonally, and (2) the duration of protection following SMC treatments.

## Methods

### Trial design

The data analysed in the present study were collected during the RTS,S-SMC individually-randomised, controlled trial conducted in Bougouni and Ouélessébougou districts, Mali and in Houndé district, Burkina Faso. Malaria transmission in these districts is intense and highly seasonal, with a peak in incidence lasting from July to November [8]. The dominant malaria parasite is *P. falciparum* (>90%) and the predominant malaria vector is *Anopheles gambiae* in each of the two study areas. A high proportion (>75%) of children sleep under a long-lasting insecticidal net (LLIN); all study children received a new LLIN upon enrolment. The trial protocol and findings have been described in detail elsewhere [4, 9].

Briefly, children were enrolled if the child would be aged 5–17 months on 1 April 2017 and randomised to one of three groups, referred to as (1) ‘SMC alone’ (Control vaccines and SMC), (2) ‘RTS,S alone’ (RTS,S/AS01_E_ vaccine and placebo SMC), and (3) ‘Combined’ intervention group (RTS,S/AS01_E_ and SMC). All SMC doses (sulfadoxine-pyrimethamine plus amodiaquine or a matching placebo, both from Guilin Pharmaceutical, China) were given as four monthly treatment courses every year, beginning in July. SMC or placebo SMC was given over 3 days, dosed according to age, as directly observed therapy, documented using a tablet PC, with QR codes on the photo ID card and drug packs to ensure each treatment was given to the correct child, and that all doses administered were recorded. All vaccine doses (RTS,S/AS01_E_ or control vaccines) were administered in April, May, and June of 2017 prior to the rainy season (July to October) and in the month of June in 2018 and 2019. RTS,S/AS01_E_ was provided by GSK, Belgium. Control vaccines without antimalarial activity consisted of three doses of rabies vaccine (RabipurR, Bavarian Nordic A/S, Denmark) in 2017, followed by annual hepatitis A vaccine (HAVRIXR, GSK, Belgium). Children were not screened for malaria infection at the time of vaccination or SMC, unless they were febrile or had other features suggestive of malaria. Children who were found to have malaria were treated according to national guidelines, offered vaccination when they had recovered and were eligible to receive the next round of SMC or placebo SMC.

### Surveillance for malaria

Project staff based in study health facilities identified and tested all suspected cases of malaria using HRP2-based Rapid Diagnostic Tests (RDTs). RDT-confirmed cases were managed with artemether-lumefantrine, following national guidelines. A case of malaria was suspected if a child presented at a health facility in the study area with fever or a history of fever within the past 48 h (or another symptom or sign suggestive of malaria) and without other symptoms that could explain the fever. A blood film was obtained from all suspected malaria cases for subsequent microscopy, read by two independent microscopists. Parasite density was estimated and discrepancies were resolved by a third reader, when necessary, using a standardised algorithm [10]. The primary outcome was microscopically-confirmed clinical malaria, defined as either measured axillary temperature ≥ 37.5°C or a history of fever within the past 48 h, and *P. falciparum* parasitemia ≥ 5000/ul, in children presenting at a study health facility. Children admitted to hospital with malaria symptoms were included in the primary outcome, if they met the above definition.

### Statistical analysis

#### Protective efficacy of RTS,S/AS01_E_ vaccine in relation to time since vaccination, among SMC recipients

**Fig. 1:**
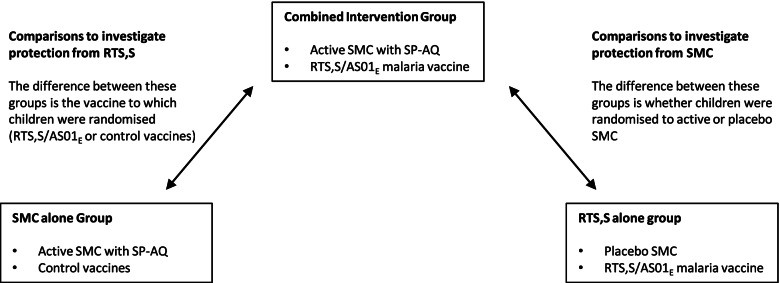
Schematic of the comparisons made in the analyses. Schematic of the comparisons made in the analyses. The analyses to estimate protection from the RTS,S/AS01_E_ malaria vaccine (presented first in the paper) compare the combined intervention group with the SMC alone group. The analyses to estimate protection from SMC (presented second in the paper) compare the combined intervention group with the RTS,S alone group

All analyses of the protective efficacy of RTS,S/AS01_E_ over time were based on a comparison of the Combined and SMC alone groups. Both groups received active SMC; the only difference between these groups was that one was randomly assigned to receive the RTS,S/AS01_E_ vaccine (the Combined group) and the other to receive control vaccines (the SMC alone group) (Fig. 1).

Protective efficacy was assessed separately for each ‘study year’, following the scheduled date for receipt of the third dose of the primary series (given in 2017), the scheduled date of the first booster (fourth dose) given in June 2018, and the scheduled date of the second booster (fifth dose) given in June 2019, including all children remaining in the SMC alone and Combined intervention groups at the time that these vaccination doses were scheduled. The analysis period in each study year began 14 days after the administration of the third dose of the primary series or 14 days after the administration of the annual booster (in order to allow for the delayed response to vaccination) until the end of the corresponding study year, including the period of low malaria transmission (31 March 2018, 2019 and 2020, for study years one, two and three, respectively). Although the underlying risk in both the SMC alone and Combined groups was reduced by SMC during the period when SMC was administered between approximately 1 and 5 months post-vaccination (Additional file 1: Fig. S1), the difference between the groups remains the receipt of RTS,S/AS01_E_ in the Combined group and the receipt of control vaccines in the SMC alone group. Consequently, assuming no interaction (this assumption is discussed later) their comparison can be interpreted as vaccine efficacy.

All passively-detected clinical malaria episodes during each analysis period were included in the analyses. To avoid double counting of events resulting from more than one health care contact, malaria episodes within 7 days of a prior episode were considered as the same event, without adjustment to person-time at risk [11].

##### Efficacy in 6-month periods

Initially, to facilitate comparisons with other malaria vaccine studies, the protective efficacy of RTS,S/AS01_E_ was estimated in 6-month periods after each dose, starting from 14 days after the third, fourth or fifth dose, using Cox regression models with a robust standard error (i.e. the Andersen-Gill extension of the Cox model), stratified by study country.

The profile of protective efficacy over time since the final dose in each study year was then investigated in more detail by comparing malaria incidence in the Combined group to that in the SMC-alone group, using three regression approaches, referred to as ‘Piecewise Cox Regression’, ‘Flexible Parametric Models’ and ‘Smoothed Schoenfeld Residuals’, as described in more detail below. Three methods were used in order to avoid the estimated profile of protection being dependent on a single set of assumptions from a particular model. In each case, country was a stratification factor, and robust standard errors were used to account for recurrent events in the same child when calculating confidence intervals. The first two methods used Stata (version 16, College Station, Texas), and the third used R (version 3.6.3).

##### Piecewise Cox regression

Person-time at risk time was stratified more finely into 90-day periods, starting from 14 days after the third, fourth or fifth doses of RTS,S/AS01_E_ (for the first, second and third year of the study, respectively). The hazard ratio (HR) comparing the Combined group to the SMC group was estimated for each 90-day period by including a term for the interaction between time period and intervention group in the Cox model. The hazard ratio was assumed to be constant within each 90-day period (this was a compromise between assuming efficacy would remain constant over each stratum and having sufficient events within each stratum that there was reasonable precision around the estimated HR). The average protective efficacy of RTS,S/AS01_E_ during each period was estimated as (1-HR), expressed as a percentage.

##### Flexible parametric models

The second approach fitted flexible parametric survival models (Royston-Parmar models) to the data using the ‘stpm2’ commands in Stata [12], which model the baseline hazard using restricted cubic splines, and allow a time-varying effect of the RTS,S/AS01_E_ vaccine (relative to control vaccines) to be estimated. Models were fitted with 1 to 10 degrees of freedom for the cubic spline, and 1 to 10 degrees of freedom for the effect of intervention group (i.e. combined group versus SMC-alone group (receipt of RTS,S/AS01_E_ vs. a control vaccine)) as a time-varying covariate. All combinations of the degrees of freedom for the cubic spline and intervention effect were fitted, i.e. 100 candidate models for each study year. The Bayesian information criterion (BIC) was used to compare models. The hazard ratio comparing the Combined group to the SMC alone group, as a function of time since vaccination, and its 95% confidence interval, were estimated from the best fitting model for each year. Time-specific efficacy, as a percentage, was calculated as (1-HR).

##### Smoothed Schoenfeld residuals

The third approach estimated the changing log hazard ratio over time since vaccination in each study year, by smoothing the scaled Schoenfeld residuals after fitting a Cox regression model, as described previously [13, 14]. The Cox models included a term for the intervention group and a robust standard error to account for between-child variability. The ‘cox.zph’ package in R was used to obtain a smooth estimate of the log hazard ratio and its 95% confidence interval, over time, and the exponentiated estimates were then used to calculate a smoothed estimate of protective efficacy over time since vaccination as (1-HR), expressed as a percentage.

#### Average protective efficacy of SMC treatments, during 30 days post-treatment, among recipients of the RTS,S/AS01_E_ vaccine

All analyses of the protective efficacy of SMC were based on comparisons of the Combined intervention group with the RTS,S/AS01_E_ alone group. Both groups received the RTS,S/AS01_E_ vaccine, but differed with respect to whether they were randomly assigned to receive active SMC or placebo SMC (Fig. 1).

The protective efficacy of SMC in the first 30 days after each SMC administration (i.e. according to the WHO recommended schedule [15]) was assessed separately for each of the 12 cycles of SP-AQ provided during the study period. The analysis was restricted to the 30-day period after each administration because children were scheduled to receive a subsequent SMC treatment after 30 days (apart from the last cycle each year). Consequently, person-time that accrued beyond 30 days after each SMC cycle was largely contributed by children who were not adherent to the schedule (which may be associated with malaria risk, i.e. subject to confounding).

For each 30-day period, all clinical malaria episodes were included starting from the date of the first daily dose of SMC (with the same rule applied as described above to avoid double counting). Hazard ratios (assumed constant within each 30-day period) were estimated from piecewise Cox regression models, stratified on study country, with a robust standard error to account for multiple episodes per child. Average protective efficacy during each period was estimated as (1-HR), expressed as a percentage.

To understand the effectiveness of SMC with different levels of adherence (including the upper limit of effectiveness with full adherence to all three daily doses), this analysis was repeated with three analysis populations, using the information on daily doses recorded by study staff who administered SMC on each day. The first analysis population, referred to as ‘Scheduled SMC’, included all children remaining in follow-up at the time of the SMC cycle, irrespective of whether any SMC was received in practice (i.e. analysis according to the randomised intervention group, provided that the child remained in the study). For children who did not receive SMC in a particular month, the median date of administration for that cycle in the same country and month, was used to define the start of the 30-day period. The second analysis population, referred to as ‘Received SMC’, included children who received at least the first daily dose of the SMC cycle, (whether or not they received the subsequent daily doses). Thirdly, the ‘Full SMC’ analysis population was restricted to children for whom all three daily doses of SMC or placebo SMC were recorded as having been successfully administered as DOT.

A pooled estimate of the average protective efficacy of SMC over 30 days post-treatment, combining the data for all 12 cycles of SMC, was also calculated for each of these three analysis populations, using a Cox model stratified by country and SMC cycle. Finally, to give an estimate of the protection provided by incomplete SMC, a pooled estimate of protective efficacy over all 12 cycles was calculated for children who received the first dose of SMC or SMC placebo in a particular month but who did not receive doses 2 and 3 (i.e. it was documented on the tablet PC that AQ was not administered on days 2 and 3).

To investigate the level of protection in the first 3 weeks post SMC (prior to the period when efficacy is thought to wane), estimates of protection in the first 21 days after each SMC cycle were also calculated for the ‘Received SMC’ population and compared to the results over the first 30 days.

#### Protective efficacy of SMC treatments in relation to time since treatment, among recipients of RTS,S/AS01_E_ vaccine

The profile of SMC protection over time is important when considering the suitability of the current monthly scheduling of SMC, and interpreting the frequent observation that, in the context of SMC programmes, a large number of malaria episodes occur shortly before the next monthly administration is due. The profile of protection was estimated over two periods: the first 30 days after SMC administration, including data from all 12 SMC cycles, and the first 60 days post-administration, including data from SMC cycles 4, 8 and 12 (the final SMC cycle each year). Figure 5 (described in more detail below) shows the number of events in this period. Since SMC was administered every 30 days during the transmission season, follow-up after the first three cycles each year is truncated by the subsequent cycle. The profile of protection was estimated up to 30 days, pooling data from all 12 SMC cycles, and adjusting for cycle. To estimate in more detail how protective efficacy varies over time since administration, avoiding the issue of censoring due to administration of the next SMC cycle, person-time and episodes after the final cycle of SMC in each year were also analysed separately, with follow-up truncated at 60 days. This was first done by pooling across all three final cycles (i.e. SMC 4, SMC 8 and SMC 12); the final cycle each year was also then analysed individually.

In the regression models described below, the specific SMC cycle was included as a 12-level categorical variable in the analysis of malaria incidence up to 30 days after all SMC cycles, and as a 3-level categorical variable in the analysis of incidence up to 60 days after SMC 4, 8 and 12.

As for the analysis of vaccine protection over time, both flexible parametric models and smoothed Schoenfeld residuals from Cox regression were used to obtain smooth estimates of the additional protective efficacy offered by SMC over time over these periods. The first of these, flexible parametric survival models, adjusted for study country and SMC cycle, with a robust variance estimator to account for multiple episodes in the same child, and models were fitted with up to 10 degrees of freedom for the baseline hazard and 10 degrees of freedom for the effect of SMC as a time-varying covariate. The Bayesian Information Criterion (BIC) was used to compare models. The hazard ratio and its 95% CI was predicted over time, with protective efficacy (1-HR, expressed as a percentage) estimated for the period of interest (30 days or 60 days).

The second method smoothed the scaled Schoenfeld residuals from a Cox model to estimate the log hazard ratio for SMC, and its confidence interval, over time. Protective efficacy was calculated from the exponentiated hazard ratio. Analyses were conducted pooling all events and person-time for the first 30 days after all SMC cycles, and for the first 60 days after the final SMC cycle each year. The Cox models included the intervention group (Combined vs. RTS,S/AS01_E_ alone), adjusted for SMC cycle as described above, included a robust standard error, and stratified on study country.

## Results

### Protective efficacy of RTS,S/AS01_E_ vaccine in relation to time since vaccination, among SMC recipients

As described above, the protective efficacy of RTS,S/AS01_E_ was estimated through comparisons of the Combined intervention group with the SMC alone group. The characteristics of study children at the time of the third priming dose, and the fourth and fifth doses (first and second boosters) are shown in Table 1. The mean age of study children was 14.4 months, 25.4 months, and 37.5 months at the time of the third, fourth, and fifth doses of vaccine, respectively.

**Table 1:** Age and sex of study children at the time of the third, fourth (first booster) and fifth (second booster) doses of RTS,S/AS01_E_ during the study period, by intervention group

	SMC alone		RTS,S alone		Combined	
	No.	%	No.	%	No.	%
**Study year 1**	***N*=1965**		***N*=1988**		***N*=1967**	
Received dose 1	1965	100	1988	100	1967	100
Received dose 2	1894	96.4	1911	96.1	1907	96.9
Received dose 3	1827	93.0	1860	93.6	1845	93.8
Mean age at dose 3 in months (SD)	14.4 (4.27)		14.5 (4.17)		14.4 (4.16)	
Sex						
Male	944/1827	51.7	965/1860	51.9	948/1845	51.4
**Study year 2**	***N*=1904**		***N*=1927**		***N*=1919**	
Received fourth dose	1798	94.4	1834	95.2	1835	95.6
Mean age at fourth dose in months (SD)	25.4 (4.28)		25.5 (4.16)		25.4 (4.19)	
Sex						
Male	931/1798	51.8	953/1834	52.0	948/1835	51.7
**Study year 3**	***N*=1847**		***N*=1882**		***N*=1873**	
Received fifth dose	1748	94.6	1776	94.4	1780	95.0
Mean age at fifth dose in months (SD)	37.5 (4.27)		37.6 (4.15)		37.5 (4.18)	
Sex						
Male	903/1748	51.7	922/1882	51.9	919/1873	51.6

For estimation of vaccine efficacy over time, the comparisons focus on the SMC alone and combined intervention groups (since the difference between these groups is that one received control vaccines and the other received RTS,S/AS01_E_)

*Abbreviation*: *RTS,S* RTS,S/AS01_E_

Efficacy in 6-month periods: Starting 14 days after the third priming dose, the overall estimated efficacy of RTS,S/AS01_E_, among SMC recipients, in the first 6 months was 75.9% (95% CI: 67.0, 82.4). Between months 6-12, this was 31.1% (95% CI: -64.2, 71.1), the wide CI reflecting the small number of events in this period. Starting 14 days after the fourth dose, the efficacy was 63.6% (95% CI: 57.2, 69.1) in the first 6 months and 61.4% (95% CI: 42.3, 74.2) between 6 and 12 months post-dose four. Starting 14 days after the fifth dose, efficacy was 60.1% (95% CI: 52.9, 66.2) in the first 6 months and 35.9% (95% CI: 0.8, 58.6) between 6 and 12 months post-dose five.

**Fig. 2:**
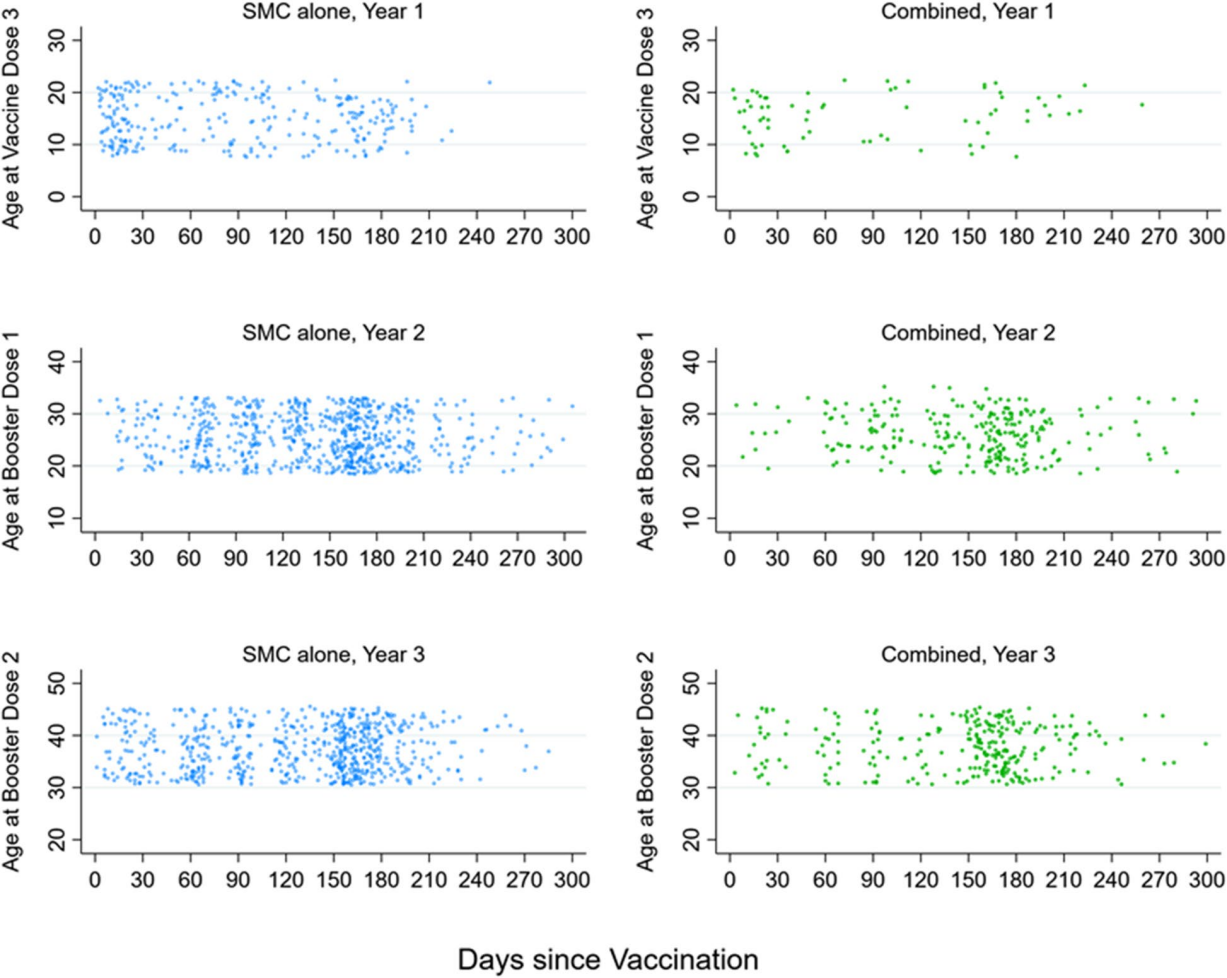
Timing of microscopically confirmed clinical malaria episodes after the final vaccination each year, among children in the SMC alone group and the combined group. Points show the timing of episodes of the primary outcome in the SMC alone group (who received SMC and control vaccines) and the Combined group (who received SMC and the RTS,S/AS01_E_ malaria vaccine), in relation to the final dose of vaccination each year (dose 3 for year 1, dose 4 for year 2, and dose 5 for year 3). These groups were compared to estimate the protective efficacy of RTS,S/AS01_E_ over time. The position relative to the y-axis indicates the age in months of the child at the time of the episode

Figure 2 shows the timing of episodes of the primary outcome by time since vaccination in the SMC alone group and Combined group, against child age. There were no marked differences by child age in any year. In all 3 years, in both groups, there were few events beyond approximately 210 days post-vaccination, reflecting the end of the malaria transmission season in the study areas (Additional file 1: Tables S1 and S2). This was particularly marked in year 1, when there were very few events after 210 days. In years two and three, both the SMC alone group and—to a lesser extent—the combined group, experienced a substantial increase in malaria cases approximately 150 days post-vaccination, reflecting the time when protection from the fourth SMC cycle waned, and there remained considerable malaria transmission in the study areas.

**Fig. 3:**
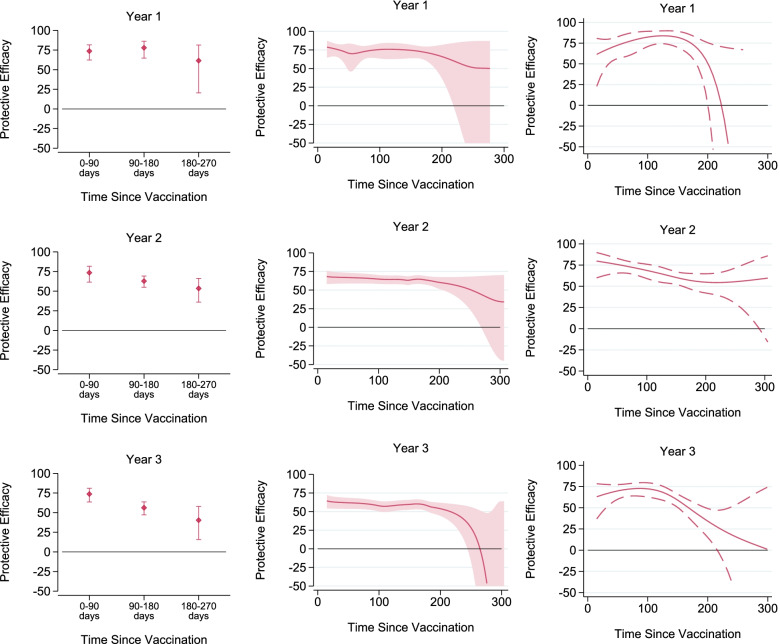
Protective efficacy of RTS,S/AS01_E_ vaccine against clinical malaria, by time since vaccination, in each year of the study, using three methods. Footnotes: Protective efficacy was estimated using three methods by comparing children randomised to receive SMC and either control vaccines (SMC alone group) or RTS,S/AS01_E_ vaccine (combined group). The left panels show results from piecewise Cox regression models. The middle panels show estimates from flexible parametric survival models. The right panels show results from Smoothed Schoenfeld Residuals from Cox regression models

Figure 3 shows the results of the regression modelling to explore vaccine efficacy over time since vaccination. The left-hand panels show the results of the piecewise Cox regression models, stratified into 90-day periods since vaccination. In all 3 years, the confidence interval widened in the third time stratum, after 180 days, reflecting the lower number of events in the subsequent dry season. The point estimate of protective efficacy remained above 60% over each stratum of the first study year (after the priming series) and remained above 50% over all strata in the second study year (after the fourth dose/first booster). In the third year (after the fifth dose/second booster) the point estimate of protective efficacy fell below 40% from 180 days onwards, but confidence intervals around the point estimate were relatively wide.

The central panels of Fig. 3 show estimates of protection over time from flexible parametric survival models. The smoothed hazard functions, cumulative hazard functions and the predicted hazard functions from the best fitting models are shown in Additional file 1: Fig. S1. In the second and third years, the flexible parametric model was able to replicate closely the cumulative baseline hazard; in the first year of the study, the fit was reasonable, but not as good. In all 3 years, the profile of protection remained relatively flat over the first 6–7 months (180–210 days) since vaccination (i.e. suggested neither very high initial efficacy, nor strong waning before the end of the transmission season). There was then a suggestion of waning efficacy from approximately 200 days onwards, in each year, but by design (due to the seasonal vaccination schedule) the period of apparent waning is also the period when malaria incidence is very low, so the width of the CI increases markedly. There was some suggestion that efficacy over the first 200 days was slightly lower after the booster doses than after the priming series, with the flat portion of the efficacy profile centred at about 75% in year 1, 65% in year 2, and 60% in year 3, although the confidence intervals overlap.

Finally, the right-hand panels of Fig. 3 show the results from smoothing the scaled Schoenfeld residuals from Cox regression models. This approach avoids having to model the baseline hazard parametrically, which is an advantage in situations—such as this one—in which the baseline hazard is complex (Additional file 1: Fig. S1) and thus does not have the same issues with model fit (in study year 1) as the flexible parametric models. The results from this analysis were consistent with the two other approaches—suggesting relatively stable efficacy over the first 6 months post-vaccination. The main difference was that the estimated efficacy fell more gradually in the period 100–200 days in study years 2 and 3 (rather than remaining stable and then falling sharply, as was suggested by the flexible parametric models). However, as for the other models, uncertainty around the estimates of additional protective efficacy increased substantially in the period beyond 200 days.

### Average protective efficacy of SMC treatments, during 30 days post-treatment, among recipients of RTS,S/AS01_E_ vaccine

**Fig. 4:**
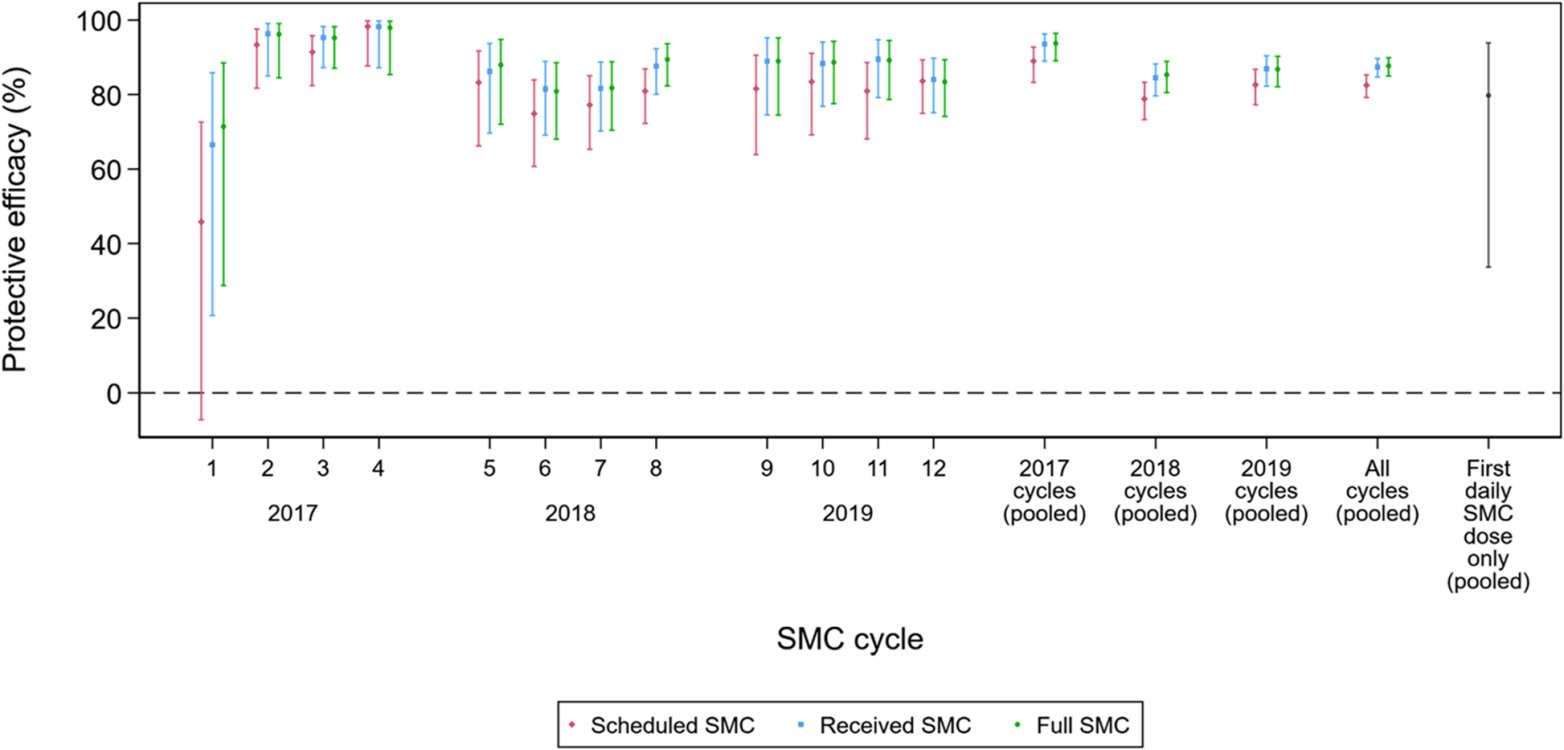
Protective efficacy of SMC in the first 30 days post-administration, among recipients of RTS,S/AS01_E_, according to adherence to SMC. Estimates of protective efficacy of SMC from Cox regression, as described in the text. SMC cycles 1, 2, 3 and 4 were administered in 2017, SMC cycles 5, 6, 7 and 8 in 2018, and SMC cycles 9, 10, 11 and 12 in 2019. ‘Scheduled SMC’ includes all children remaining in follow-up at the time of the SMC cycle, irrespective of whether any SMC was received. ‘Received SMC’, includes only children who received at least the first daily dose of the SMC cycle, but who may not have received subsequent doses. ‘Full SMC’ includes only children for whom all 3 daily doses of SMC or placebo SMC were successfully administered and documented. ‘First daily SMC dose only (pooled)’ includes only children who were confirmed to have received the day 1 dose of SP+AQ, but not the day 2 or day 3 doses of AQ, pooled across all 12 cycles given during the study period

As described above, the protective efficacy of SMC was estimated through comparisons of the Combined intervention group with the RTS,S alone group. The number of children who were scheduled to receive SMC, received at least one dose of SMC, and received all three daily doses (Scheduled SMC, Received SMC and Full SMC, respectively) is shown in Additional file 1: Table S3. The average protective efficacy in the first 30 days after each SMC cycle, estimated by Cox regression, is shown for these three analysis populations in Fig. 4. The number of children experiencing malaria in the 30 days after SMC 1 was low (24 in total from the RTS,S/AS01_E_ alone group, and 13 in total from the combined group), and thus estimates of protective efficacy are imprecise. From SMC 2 onwards, the additional protective efficacy of SMC remained high over the remaining 11 cycles, with the lowest point estimates of protective efficacy of 74.9% in children scheduled to receive SMC (irrespective of whether they received SMC), 81.5% in children who received at least one dose of SMC, and 80.9% in children who received all 3 daily SMC doses. Pooling across all 12 SMC cycles, the point estimate of the additional protective efficacy offered by SMC, over RTS,S/AS01_E_ alone, was 82.5%, 87.4% and 87.7% in children who were scheduled to receive SMC, received at least one dose, and received all three doses, respectively. Over the 12 cycles, pooling children who received only SP-AQ on the first day, the protective efficacy was 79.8%, although the 95% CI ranged from 33.7 to 93.8%, as relatively few children were not adherent in the trial.

Exploratory analysis of protection, stratifying follow-up to include only episodes occurring in the first 21 days after receipt of SMC indicated a higher level of protection than over the first 30 days. However, even in this shorter period, closer to the time of SMC administration, protection was not complete after most of the SMC cycles (Additional file 1: Fig. S2).

### Protective efficacy of SMC treatments in relation to time since treatment, among recipients of RTS,S/AS01_E_ vaccine

**Fig. 5:**
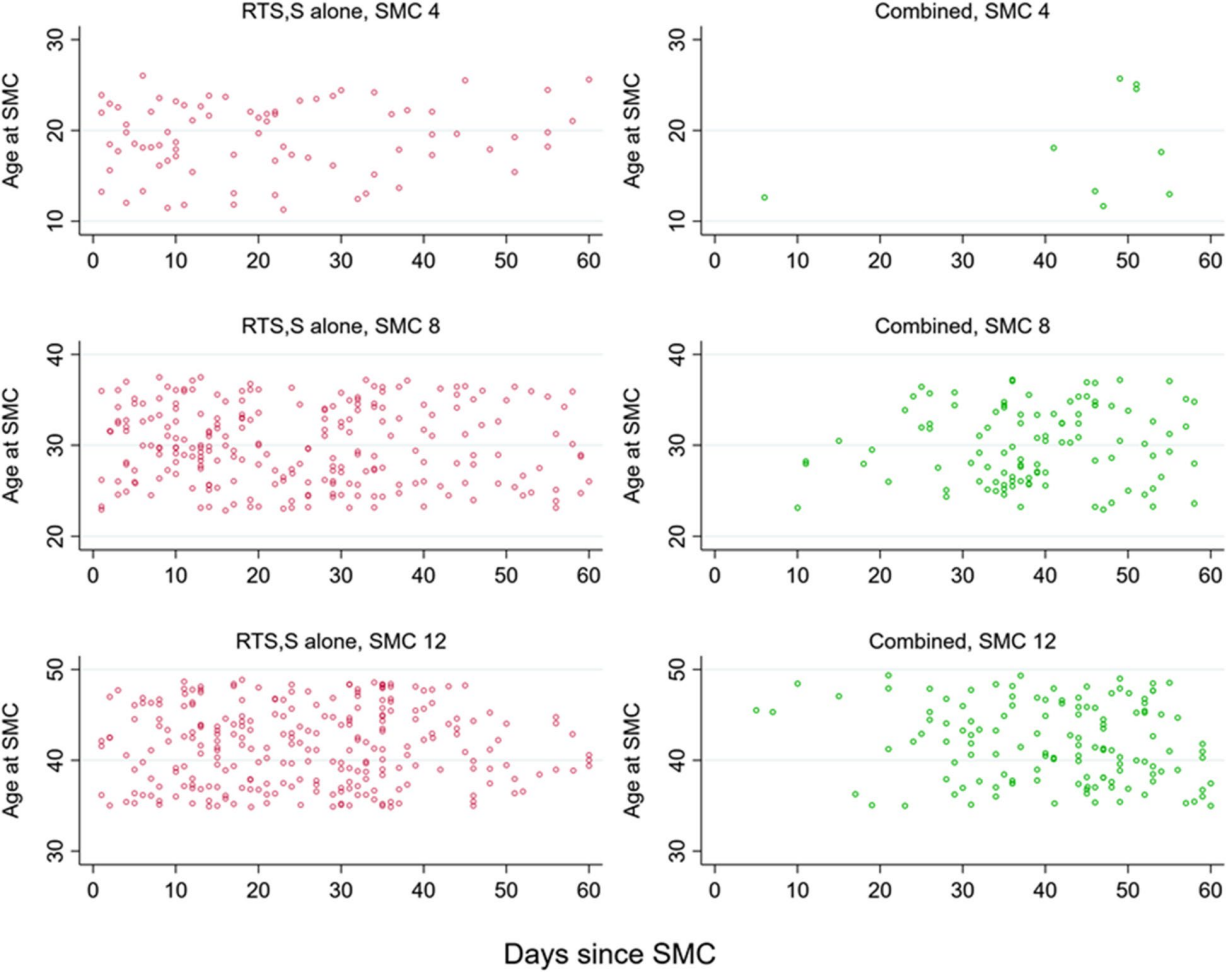
Timing of microscopically confirmed clinical malaria episodes after the final SMC cycle in each year of the study, among recipients of RTS,S/AS01_E_, randomised to receive either placebo SMC (RTS,S alone group) or active SMC (Combined group). Points show the timing of episodes of the primary outcome in the RTS,S/AS01_E_ alone group (who received placebo SMC and the RTS,S vaccine) and the Combined group (who received active SMC and the RTS,S vaccine), in relation to the final SMC cycle administered each year (SMC 4 in year 1, SMC 8 in year 2, and SMC 12 in year 3). The position relative to the y-axis indicates the age in months of the child at the time of the episode

Figure 5 shows the timing of events by time since the final SMC cycle each year (i.e. SMC 4, SMC 8 and SMC 12) in the RTS,S/AS01_E_ alone group and the Combined group, according to child age. There were no marked differences in incidence by age. There were very few events after SMC 4 in the Combined group, reflecting the lower incidence in 2017 (when study children were younger), but also the later timing of the SMC cycles with respect to the transmission season, which resulted in SMC protection persisting until transmission had declined to low levels. After SMC 8 and SMC 12, the number of malaria episodes in the Combined group increased markedly after approximately 30 days.

**Fig. 6:**
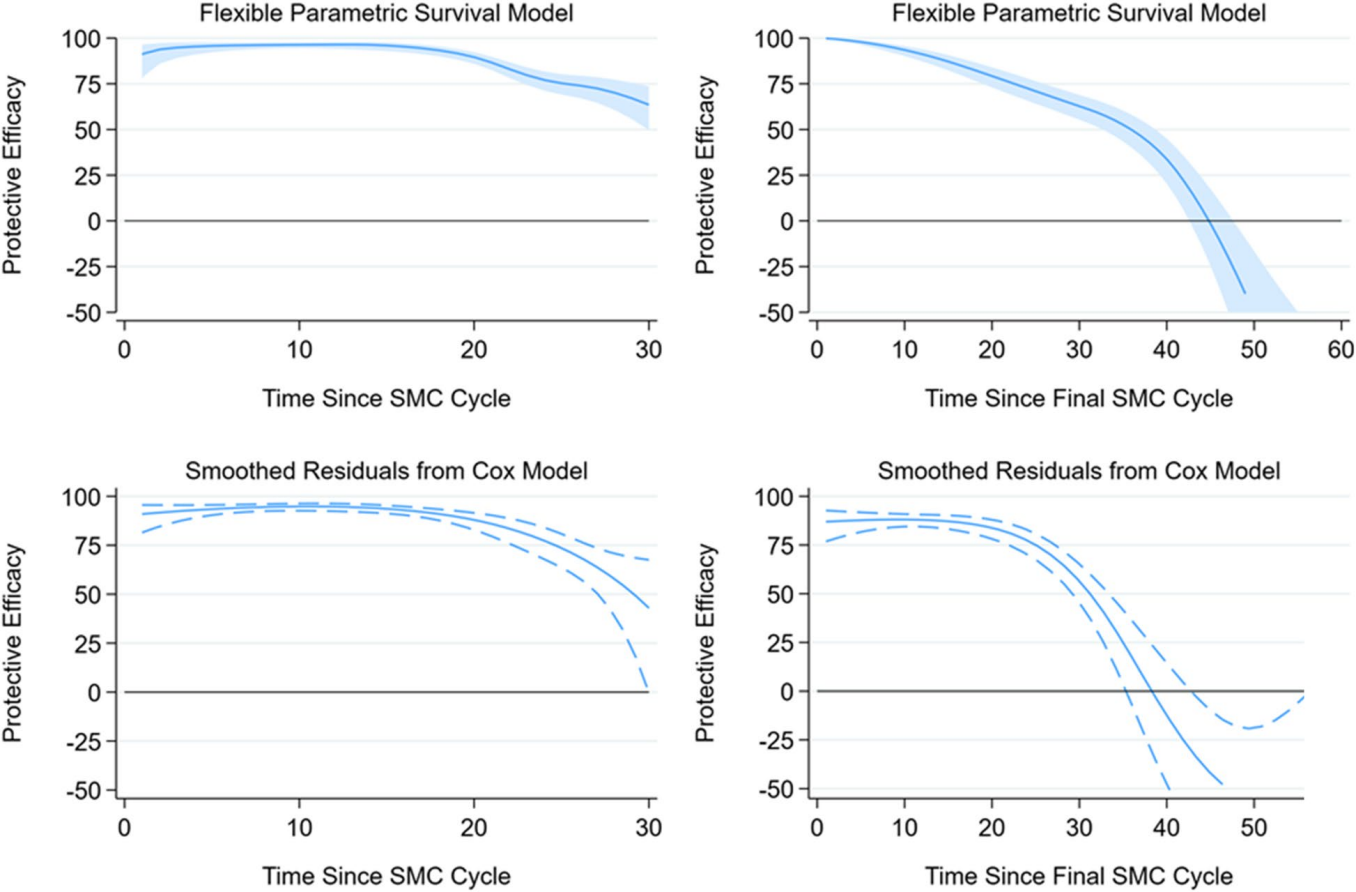
Profile of protective efficacy of SMC against clinical malaria, among recipients of RTS,S/AS01_E_. Footnotes: Protective efficacy of SMC against clinical malaria over time, among children randomised to receive the RTS,S/AS01_E_ malaria vaccine. Results are presented up to 30 days post-SMC (left panels), combining data from all 12 SMC cycles; and up to 60 days post-SMC (right panels), combining data from the final SMC cycle in each year of the study. The top panels show results from flexible parametric survival models, and the bottom panels show results from smoothed Schoenfeld residuals from Cox regression models

Figure 6 shows the smooth estimates of additional protective efficacy offered by SMC over time, using Flexible Parametric Models and Smoothed Schoenfeld Residuals from Cox models. Combining data from all 12 cycles, in order to estimate protection over the first 30 days, the protective efficacy was very high in the first 2 weeks and then began to wane, with the loss of protection most marked in the last 7–10 days of the 30-day interval between SMC cycles. This finding was consistent with both flexible parametric survival models (Fig. 6, top left) and estimates based on smoothed Cox residuals (Fig. 6, bottom left).

Combining data from the final SMC cycle each year, in order to estimate protection over 60 days, protective efficacy was observed to decline slowly initially, and then more rapidly after 30 days. The estimated time at which the additional protective efficacy was completely lost appeared to be at around 45 days after SMC administration using Flexible Parametric Models, and slightly shorter (at about 38 days) using Smoothed Cox Residuals. Similar results and a similar estimate of protection at 30 days were obtained when these models were fitted separately to data from the last SMC cycle in each individual year (Additional file 1: Fig. S3), although the confidence intervals around the estimates were wider, particularly after SMC 4.

## Discussion

The estimates of protective efficacy provided by RTS,S/AS01_E_ among SMC recipients in this study during the first 12 months after the third priming dose of RTS,S/AS01_E_ are consistent with the estimated efficacy of RTS,S/AS01_E_ against placebo in the 12 months post-vaccination in the phase 3 trial [16], in which children did not receive any SMC. The average efficacy in the first 6 months after dose 3 in the RTS,S/AS01_E_ phase 3 trial was estimated at 67.6% (95% CI: 63.8 to 71.0), and 38.9% (95% CI: 36.3 to 44.0) between 6 and 12 months [17]; the comparable estimates in these two periods in our study were 75.9% (95% CI: 67.0, 82.4) and 29.7% (95% CI: − 51.9, 67.5), respectively. It is logical that our study design, which aligned peak vaccine efficacy with the season of peak malaria incidence, would emphasise the period of highest protection offered by RTS,S/AS01_E_ and thus obtain slightly higher efficacy in the initial period post-vaccination, than in the earlier trial in which vaccination was age-based rather than seasonally-targeted. The average efficacy of RTS,S/AS01_E_ in our study is also comparable with a recent phase 2b trial of the R21/Matrix M vaccine, delivered largely before the rainy season in Nanoro, Burkina Faso [18], in which protective efficacy was 77% (95% CI: 67 to 84) over the first 6 months after administration.

The profile of protection provided by RTS,S/AS01_E_ over time after vaccination, estimated with three different methods, indicates that protection remains at a relatively high level over the first 6 months post-vaccination in each study year (i.e. over the remaining period of malaria risk in these seasonal transmission settings). This was observed for the primary series, and also for the fourth and fifth doses (first and second boosters), which supports the use of seasonal booster doses of RTS,S/AS01_E_. All three methods indicated a possible decline in efficacy in the period beyond 6 months post-vaccination, and all three methods suggested that this was most marked in the third year of the study. The finding of declining efficacy is compatible with anti-CSP antibody responses, which had declined to low levels prior to the fourth and fifth doses of RTS,S/AS01_E_ [19]. A 2-year extension to the RTS,S-SMC trial will enable assessment of the protection (including the duration of protection) offered by the third and fourth booster doses of RTS,S/AS01_E_ (six and seventh doses), and assessment of whether efficacy continues to decline with successive doses. However, a feature of our study design (with seasonal administration of the vaccine) is that relatively few cases of malaria occur in the period 6–12 months post-vaccination, since this period falls in the dry season. Consequently, there is considerable uncertainty around the estimated protective efficacy beyond 6 months, and the precise extent to which efficacy declines is not clear. The public health importance of any drop in efficacy during this period is likely to be relatively small, due to the small number of cases (Additional file 1: Tables S1 and S2).

The estimated protective efficacy provided by SMC, among recipients of RTS,S/AS01_E_, is consistent with results from earlier SMC studies in unvaccinated children, in which efficacy was estimated to be about 74% over a transmission season [20] and 88% over the first 28 days [2]. The profile of protection over time was also very similar to estimates from an earlier placebo-controlled trial of SMC (previously referred to as intermittent preventive treatment in children, IPTc) [21–23] (Additional file 1: Fig. S4). The high level of protection offered by each monthly cycle of SMC is compatible with the high curative efficacy of SP-AQ in the study area [4], and the current low prevalence of AQ and SP resistance markers in Burkina Faso and Mali [3, 24]. Apart from the low and imprecise estimate of protective efficacy offered by SMC at the first SMC cycle in 2017, which was affected by a very low number of events, efficacy appeared to be slightly higher in the first year of the study. It is possible that this reflects the age profile of the cohort, resulting in a higher dosage of SP-AQ by weight among the cohort in 2017 than in later years. Dosing in the study was by age, with children above 12 months of age receiving a paediatric dose (double the infant dose). In 2017, the median age of the cohort was 15 months, i.e. just above the threshold at which the dose was doubled, whereas the median age was 26 months and 38 months in the latter 2 years, respectively. All daily doses were supervised, so adherence was very high, but results from the small number of children with incomplete adherence to the 3-day SMC regimen suggested efficacy of the first day was still high, consistent with earlier studies with shorter-acting regimens alongside SP [25, 26] or SP monotherapy [27, 28].

The profile of protective efficacy over time after SMC shows two key features. Firstly, the efficacy of SP-AQ does not reach 100% even in the initial period post-administration, and cases of malaria still occur among SMC recipients within this period. Secondly, there is a marked fall in efficacy over the latter part of each monthly cycle, from approximately day 21 onwards. This result, consistent with earlier placebo-controlled studies of SMC [23], indicates the importance of preserving the delivery interval for SMC at a maximum of 28 days. This also explains the observation in our study, and elsewhere, that cases among SMC recipients tend to occur in the days leading up to the subsequent SMC cycle. Taken together, these two findings (that protection does not reach 100%, and wanes within the monthly interval between administration) highlight limitations in the protection offered by SMC and show the potential utility of vaccination where SMC is deployed to address these limitations. The potential reduction in burden among SMC recipients that vaccination could offer is important because the incidence of episodes of clinical malaria in the SMC alone group, even in the context of very high adherence to directly-observed SMC, was approximately 300 per 1000 person-years at risk over the study period overall. In addition to its advantage in providing protection outside the SMC period (through its longer duration of protection) vaccination would also improve protection against malaria during the SMC period, particularly at times when protection offered by SMC is not at its maximum. This may have the practical advantage of reducing the number of children that cannot receive SMC at the scheduled time each month because they have clinical malaria and are treated with an effective malaria combination (i.e. vaccination may increase the percentage of children who can receive SMC, as observed in the recent trial [4]).

The three methods used here have different advantages and disadvantages. Piecewise Cox models estimate the average protection in specified time periods. Flexible parametric models estimate efficacy as a smoothly varying function over time, but are less well suited to capturing abrupt changes in efficacy or the baseline hazard (which is the case for analyses using SMC as the reference group). The approach using smoothed Cox residuals avoids this limitation, as the baseline hazard is not modelled. The consistency of results using the three methods provides reassurance that the estimated profile of protection over time is not strongly dependent on a specific set of model assumptions.

A key limitation is that the study design did not include a group who received neither intervention, so it is not possible to formally assess the evidence for an interaction between SMC and RTS,S/AS01_E_. However, because the efficacy of RTS,S/AS01_E_ was consistent with the phase 3 trial, and the efficacy of SMC consistent with previous studies, an adverse interaction seems unlikely and there was no evidence of differences in anti-circumsporozoite protein (anti-CSP) responses to the RTS,S/AS01_E_ vaccine among children who received active SMC or placebo SMC [19]. Since this analysis focuses on cases that were detected passively, it is possible that some cases of malaria that did not result in care-seeking by the caregiver were not captured. A further limitation when comparing results from this study with other vaccination trials (and between trials of malaria vaccines more generally) is that the timing of vaccination in relation to the peak transmission season, the length of the transmission season, and transmission intensity are all potential confounding factors affecting between-study comparisons. Ideally, comparisons between studies should be based on the profile of efficacy over time, adjusted for transmission intensity (i.e. the primary effect [29]), rather than average efficacy, or estimates based on the total effect. Finally, since there are relatively few cases beyond 6 months after the final dose each year, it is unclear how efficacy changes beyond this point, making it unclear to what extent this would increase risk in children if they were to be vaccinated earlier in the year.

A possible limitation of the separate analyses in each year (both for estimates of the protection from RTS,S/AS01_E_ and protection from SMC) is that these include only children who remained in follow-up, which is a sub-set of the group of children who were originally randomised to the study arms. Loss to follow-up was quite low, at 11% overall over the 3-year period of the trial, and very similar between treatment arms (11.0%, 11.4% and 10.8% in the SMC alone, RTS,S/AS01_E_ alone and Combined intervention groups, respectively). The incidence of malaria in the second and third years of the study may be influenced by the ageing of the study cohort with time, and by the different prior experiences of malaria up to that point (which is influenced by the intervention group to which children were randomised). It is possible that estimates of protective efficacy after doses 4 and 5 of the RTS,S/AS01_E_ vaccine, for example, are conservative because the reference group (SMC alone), may have acquired more protective immunity through exposure to malaria than the Combined intervention group.

Comparing the profile of additional protection offered by these two interventions, it is apparent that the peak efficacy of RTS,S/AS01_E_ in the period immediately after administration is not as high as the protective efficacy immediately after SMC. This suggests that—assuming similar coverage with the two approaches—an SMC programme that provided monthly cycles throughout the entire transmission season would provide a slightly higher level of protection than RTS,S/AS01_E_ alone over the same period. The fact that SMC was not superior in the trial, despite high coverage of both interventions, combined with the high incidence when protection offered by SMC had waned in the early months of the dry season likely reflects a four-cycle SMC programme being used in areas where the epidemiology requires at least 5 months of SMC [8]. However, the standard of care in the Burkina Faso study area has changed, with the national malaria control programme switching to a five-cycle SMC programme in some areas including the Houndé district in 2021.

Profiles of protective efficacy over time will be needed to select optimum dose timings for the RTS,S/AS01_E_ vaccine and for SMC. Alternative delivery strategies for malaria vaccines (age-based or calendar-based vaccination) will differ with respect to the time of year, and the age range, when children can receive their vaccine doses and are best protected. Understanding the optimum strategy will require consideration of the profile of protective efficacy over time in relation to the timing and duration of the transmission season, as well as the coverage that can be achieved in practice. Use of the combination of vaccination and SMC may increase the percentage of the population that has access to at least one effective intervention. Modelling exercises could also help to understand how the combined intervention of SMC and malaria vaccination might compare with other available control options. For example, the effectiveness of SMC itself may be improved by increasing the number of monthly courses (as discussed above) or extending the age range to which it is administered. Vector control may be improved through more effective LLIN and/or new tools. This could have implications for the absolute impact of adding malaria vaccination to SMC.

## Conclusions

The efficacy of both interventions was highest immediately post-administration. The peak efficacy of SMC was higher than for RTS,S/AS01_E_, but waned faster (over 3–4 weeks, versus more than 6 months for RTS,S/AS01_E_). Quantifying these key differences between these interventions may help to optimise scheduling of SMC, malaria vaccination and their combination in areas of seasonal transmission with differing malaria epidemiology, and where different vaccine delivery systems are available. If optimisation of the combination of SMC and malaria vaccination can be achieved, it could have a marked impact on the malaria burden in areas of intense and seasonal malaria transmission.

## Supplementary Information

**Additional file 1: Table S1.** Number of clinical malaria episodes by time since vaccination in each year of the study, using 90-day periods (as used in the Piecewise Cox regression models). **Table S2.** Number of clinical malaria episodes by time since vaccination in each year of the study, using 60-day periods (not used in the Piecewise Cox regression models, but provided to show the declining incidence of malaria further into the dry season). **Table S3.** Number and percentage of children who were scheduled to receive SMC, received SMC, and received all daily SMC doses (full SMC) over the course of the study. **Figure S1.** Observed hazard function and Cumulative hazard function, and the fitted cumulative hazard and estimated protective efficacy from flexible parametric survival models used to estimate vaccine efficacy in each year of the study. **Figure S2.** Protective Efficacy of SMC in the first 21 days and first 30 days after SMC received, by cycle. **Figure S3.** Protective Efficacy by time since the final SMC cycle in each year. **Figure S4.** Comparison of the SMC protective efficacy profile obtained in this study with the profile estimated for an earlier placebo-controlled trial of SMC.

## Data Availability

Data will be archived on the LSHTM Data Compass system (http://datacompass.lshtm.ac.uk/), an Open Archival Information System (OAIS)-compliant data repository operated by the institution. The data will be assigned a Digital Object Identifier (DOI). A Data Access Group will review requests to share the archived data. A data agreement document will be provided, and the requestor must concur with the restrictions in this agreement to ensure that participants’ privacy is maintained and that the data are only used for appropriate research purposes. In April 2020, study children completing the present trial were re-enrolled into an extension study for an additional 2 years. Data from the first 3 years of the study, used in these analyses, will be made available in November 2022, when the extension study has been completed.

## Abbreviations

BIC: Bayesian information criterion
CI: Confidence interval
DOT: Directly observed therapy
DSMB: Data Safety and Monitoring Board
HR: Hazard ratio
IPTc: Intermittent preventive treatment in children
RDTs: Rapid diagnostic tests
SMC: Seasonal malaria chemoprevention
SP-AQ: Sulfadoxine-pyrimethamine plus amodiaquine
WHO: World Health Organisation
