# Supplementary material for: The duration of protection against clinical malaria provided by the combination of seasonal RTS,S/AS01E vaccination and seasonal malaria chemoprevention versus either intervention given alone
Source: BMC Med. 2022 Oct 7;20:352. doi: 10.1186/s12916-022-02536-5 (PMC9540742; doi:10.1186/s12916-022-02536-5)
Supplement: Supplementary file 1 — Additional file 1: Table S1. Number of clinical malaria episodes by time since vaccination in each year of the study, using 90-day periods (as used in the Piecewise Cox regression models). Table S2. Number of clinical malaria episodes by time since vaccination in each year of the study, using 60-day periods (not used in the Piecewise Cox regression models, but provided to show the declining incidence of malaria further into the dry season). Table S3. Number and percentage of children who were scheduled to receive SMC, received SMC, and received all daily SMC doses (full SMC) over the course of the study. Figure S1. Observed hazard function and Cumulative hazard function, and the fitted cumulative hazard and estimated protective efficacy from flexible parametric survival models used to estimate vaccine efficacy in each year of the study. Figure S2. Protective Efficacy of SMC in the first 21 days and first 30 days after SMC received, by cycle. Figure S3. Protective Efficacy by time since the final SMC cycle in each year. Figure S4. Comparison of the SMC protective efficacy profile obtained in this study with the profile estimated for an earlier placebo-controlled trial of SMC. [file 12916_2022_2536_MOESM1_ESM.docx]

**Additional File 1. Supplemental Tables and Figures**

**Table S1**. Number of clinical malaria episodes by time since vaccination in each year of the study, using 90-day periods (as used in the Piecewise Cox regression models).

| Study Year | Interval* | Group | Person-years at Risk | Events | Rate per 1000 person-years (95% CI) |
| --- | --- | --- | --- | --- | --- |
| Year 1 | 1) 0-90 days | SMC alone | 479.1 | 148 | 308.9 (262.9, 362.9) |
| (Primary Series) |  | Combined | 481.7 | 39 | 81.0 (59.2, 110.8) |
|  | 2) 90-180 days | SMC alone | 474.6 | 99 | 208.6 (171.3, 254.0) |
|  |  | Combined | 476.9 | 22 | 46.1 (30.4, 70.1) |
|  | 3) 180-270 days | SMC alone | 455.8 | 26 | 57.0 (38.8, 83.8) |
|  |  | Combined | 458.5 | 10 | 21.8 (11.7, 40.5) |
|  |  |  |  |  |  |
| Year 2 | 1) 0-90 days | SMC alone | 462.9 | 150 | 324.1 (276.1, 380.3) |
| (Fourth Dose) |  | Combined | 469.4 | 40 | 85.2 (62.5, 116.2) |
|  | 2) 90-180 days | SMC alone | 459.3 | 406 | 883.9 (802.0, 974.2) |
|  |  | Combined | 466.4 | 153 | 328.0 (280.0, 384.4) |
|  | 3) 180-270 days | SMC alone | 450.6 | 132 | 292.9 (247.0, 347.4) |
|  |  | Combined | 457.9 | 62 | 135.4 (105.6, 173.7) |
|  |  |  |  |  |  |
| Year 3 | 1) 0-90 days | SMC alone | 448.6 | 192 | 428.0 (371.6, 493.1) |
| (Fifth Dose) |  | Combined | 454.7 | 51 | 112.2 (85.2, 147.6) |
|  | 2) 90-180 days | SMC alone | 439.6 | 355 | 807.5 (727.8, 896.1) |
|  |  | Combined | 446.4 | 158 | 353.9 (302.8, 413.6) |
|  | 3) 180-270 days | SMC alone | 427.5 | 91 | 212.8 (173.3, 261.4) |
|  |  | Combined | 432.6 | 55 | 127.1 (97.6, 165.6) |

* time scale begins 14 days after Dose 3 (Year 1), or 14 days after the annual seasonal booster (Year 2 and 3).

**Table S2**. Number of clinical malaria episodes by time since vaccination in each year of the study, using 60-day periods (not used in the Piecewise Cox regression models, but provided to show the declining incidence of malaria further into the dry season).

| Study Year | Interval* | Group | Person-years at Risk | Events | Rate per 1000 person-years (95% CI) | |
| --- | --- | --- | --- | --- | --- | --- |
| Year 1 | 1) 0-60 days | SMC alone | 320.0 | 115 | 359.4 (299.4, 431.5) |  |
| (Primary Series) |  | Combined | 321.5 | 36 | 112.0 (80.8, 155.2) |  |
|  | 2) 60-120 days | SMC alone | 317.7 | 63 | 198.3 (154.9, 253.8) |  |
|  |  | Combined | 319.9 | 11 | 34.4 (19.0, 62.1) |  |
|  | 3) 120-180 days | SMC alone | 316.0 | 69 | 218.3 (172.4, 276.4) |  |
|  |  | Combined | 317.2 | 14 | 44.1 (26.1, 74.5) |  |
|  | 4) 180-240 days | SMC alone | 314.2 | 25 | 79.6 (53.8, 117.8) |  |
|  |  | Combined | 315.9 | 9 | 28.5 (14.8, 54.8) |  |
|  | 5) 240-300 days | SMC alone | 145.5 | 1 | 6.87 (0.968, 48.8) |  |
|  |  | Combined | 146.2 | 1 | 6.84 (0.963, 48.5) |  |
|  |  |  |  |  |  |  |
| Year 2 | 1) 0-60 days | SMC alone | 309.1 | 63 | 203.8 (159.2, 260.9) |  |
| (Fourth Dose) |  | Combined | 313.1 | 14 | 44.7 (26.5, 75.5) |  |
|  | 2) 60-120 days | SMC alone | 307.2 | 173 | 563.1 (485.1, 653.6) |  |
|  |  | Combined | 312.2 | 54 | 173.0 (132.5, 225.9) |  |
|  | 3) 120-180 days | SMC alone | 305.9 | 320 | 1046.1 (937.6, 1167.3) |  |
|  |  | Combined | 310.5 | 125 | 402.5 (337.8, 479.7) |  |
|  | 4) 180-240 days | SMC alone | 302.8 | 117 | 386.4 (322.4, 463.2) |  |
|  |  | Combined | 307.3 | 56 | 182.2 (140.2, 236.8) |  |
|  | 5) 240-300 days | SMC alone | 291.9 | 26 | 89.1 (60.6, 130.8) |  |
|  |  | Combined | 297.6 | 12 | 40.3 (22.9, 71.0) |  |
|  |  |  |  |  |  |  |
| Year 3 | 1) 0-60 days | SMC alone | 299.8 | 105 | 350.3 (289.3, 424.1) |  |
| (Fifth Dose) |  | Combined | 303.9 | 32 | 105.3 (74.5, 148.9) |  |
|  | 2) 60-120 days | SMC alone | 296.8 | 150 | 505.4 (430.7, 593.2) |  |
|  |  | Combined | 301.1 | 41 | 136.2 (100.3, 184.9) |  |
|  | 3) 120-180 days | SMC alone | 291.6 | 292 | 1001.3 (892.8, 1123.0) |  |
|  |  | Combined | 296.2 | 136 | 459.2 (388.1, 543.2) |  |
|  | 4) 180-240 days | SMC alone | 286.2 | 82 | 286.5 (230.8, 355.8) |  |
|  |  | Combined | 289.5 | 49 | 169.2 (127.9, 223.9) |  |
|  | 5) 240-300 days | SMC alone | 266.9 | 12 | 45.0 (25.5, 79.2) |  |
|  |  | Combined | 270.1 | 10 | 37.0 (19.9, 68.8) |  |

* time scale begins 14 days after Dose 3 (Year 1), or 14 days after the annual seasonal booster (Year 2 and 3).

**Table S3.** Number and percentage of children who were scheduled to receive SMC, received SMC, and received all daily SMC doses (full SMC) over the course of the study.

|  | **SMC alone** | | **RTSS alone** | | **Combined** | |
| --- | --- | --- | --- | --- | --- | --- |
| **Year 1** | **N** | **%** | **N** | **%** | **N** | **%** |
| Scheduled to receive SMC | 1965 | 100 | 1988 | 100 | 1967 | 100 |
|  |  |  |  |  |  |  |
| Received SMC 1 | 1684 | 85.7 | 1801 | 90.6 | 1774 | 90.2 |
| Full SMC at SMC 1 | 1631 | 83.0 | 1753 | 88.2 | 1721 | 87.5 |
|  |  |  |  |  |  |  |
| Received SMC 2 | 1767 | 89.9 | 1819 | 91.5 | 1791 | 91.1 |
| Full SMC at SMC 2 | 1700 | 86.5 | 1762 | 88.6 | 1746 | 88.8 |
|  |  |  |  |  |  |  |
| Received SMC 3 | 1727 | 87.9 | 1726 | 86.8 | 1758 | 89.4 |
| Full SMC at SMC 3 | 1658 | 84.4 | 1673 | 84.2 | 1703 | 86.6 |
|  |  |  |  |  |  |  |
| Received SMC 4 | 1728 | 87.9 | 1762 | 88.6 | 1767 | 89.8 |
| Full SMC at SMC 4 | 1660 | 84.5 | 1704 | 85.7 | 1704 | 86.6 |
|  |  |  |  |  |  |  |
| **Year 2** | **N** | **%** | **N** | **%** | **N** | **%** |
| Scheduled to receive SMC | 1904 | 100 | 1927 | 100 | 1919 | 100 |
|  |  |  |  |  |  |  |
| Received SMC 5 | 1705 | 89.5 | 1754 | 91.0 | 1752 | 91.3 |
| Full SMC at SMC 5 | 1674 | 87.9 | 1725 | 89.5 | 1720 | 89.6 |
|  |  |  |  |  |  |  |
| Received SMC 6 | 1636 | 85.9 | 1717 | 89.1 | 1718 | 89.5 |
| Full SMC at SMC 6 | 1590 | 83.5 | 1683 | 87.3 | 1685 | 87.8 |
|  |  |  |  |  |  |  |
| Received SMC 7 | 1631 | 85.7 | 1684 | 87.4 | 1707 | 89.0 |
| Full SMC at SMC 7 | 1600 | 84.0 | 1667 | 86.5 | 1679 | 87.5 |
|  |  |  |  |  |  |  |
| Received SMC 8 | 1601 | 84.1 | 1671 | 86.7 | 1682 | 87.6 |
| Full SMC at SMC 8 | 1564 | 82.1 | 1651 | 85.7 | 1658 | 86.4 |
|  |  |  |  |  |  |  |
| **Year 3** | **N** | **%** | **N** | **%** | **N** | **%** |
| Scheduled to receive SMC | 1847 | 100 | 1882 | 100 | 1873 | 100 |
|  |  |  |  |  |  |  |
| Received SMC 9 | 1680 | 91.0 | 1727 | 91.8 | 1727 | 92.2 |
| Full SMC at SMC 9 | 1667 | 90.3 | 1710 | 90.9 | 1708 | 91.2 |
|  |  |  |  |  |  |  |
| Received SMC 10 | 1644 | 89.0 | 1687 | 89.6 | 1700 | 90.8 |
| Full SMC at SMC 10 | 1635 | 88.5 | 1669 | 88.7 | 1684 | 89.9 |
|  |  |  |  |  |  |  |
| Received SMC 11 | 1642 | 88.9 | 1677 | 89.1 | 1687 | 90.1 |
| Full SMC at SMC 11 | 1635 | 88.5 | 1666 | 88.5 | 1681 | 89.7 |
|  |  |  |  |  |  |  |
| Received SMC 12 | 1667 | 90.3 | 1692 | 89.9 | 1703 | 90.9 |
| Full SMC at SMC 12 | 1663 | 90.0 | 1686 | 89.6 | 1698 | 90.7 |

The number and percentage of children still in follow-up at the beginning of each study year who were scheduled to receive SMC, who received the first daily dose of SMC (Received SMC), and who received all SMC doses (Full SMC), for each of the 12 SMC courses administered over the study period. For estimation of SMC efficacy over time, the comparisons focus on the RTS,S alone and Combined intervention groups since the difference between these groups is that one received SMC active and one received placebo SMC.

**Figure S1**. Observed hazard function and Cumulative hazard function, and the fitted cumulative hazard and estimated protective efficacy from flexible parametric survival models used to estimate vaccine efficacy in each year of the study.


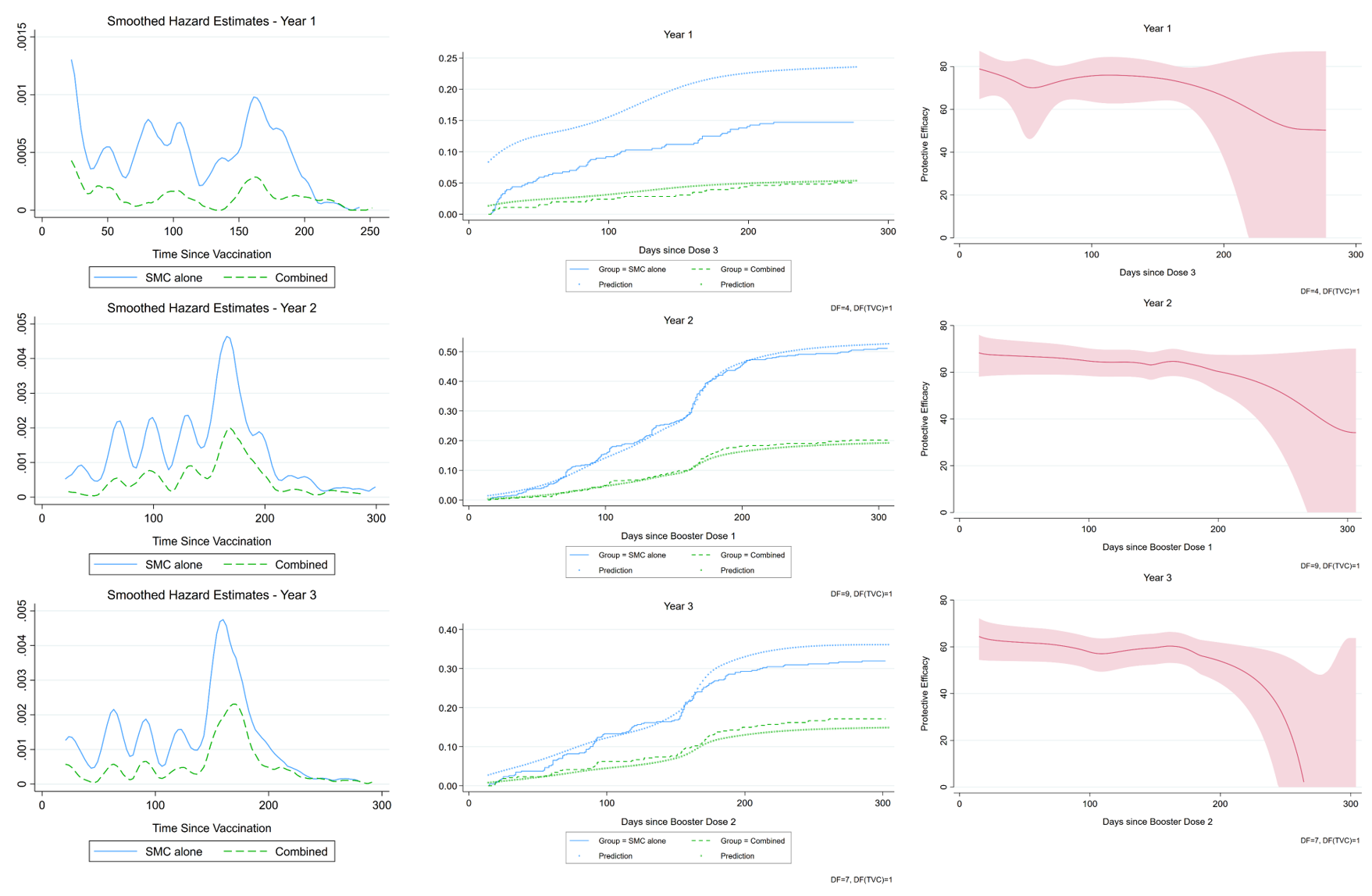


Footnote. The rows show results for the additional protective efficacy of RTS,S/AS01_E_ among SMC recipients in year 1, year 2 and year 3 of the study respectively (specifically, the period starting 14 days after dose 3, dose 4 and dose 5 of RTS,S). The left-hand panels show the smoothed hazard function in the SMC alone (solid blue line) and the Combined intervention group (dashed green line). The middle panels show the observed cumulative hazard (solid lines), and the predicted cumulative hazard from the fitted flexible parametric survival model (dotted lines). The right-hand panels show the estimated protective efficacy over time using the flexible parametric survival models (red line) and the 95% CI (red shaded area).

**Figure S2**. Protective Efficacy of SMC in the first 21 days and first 30 days after SMC received, by cycle.


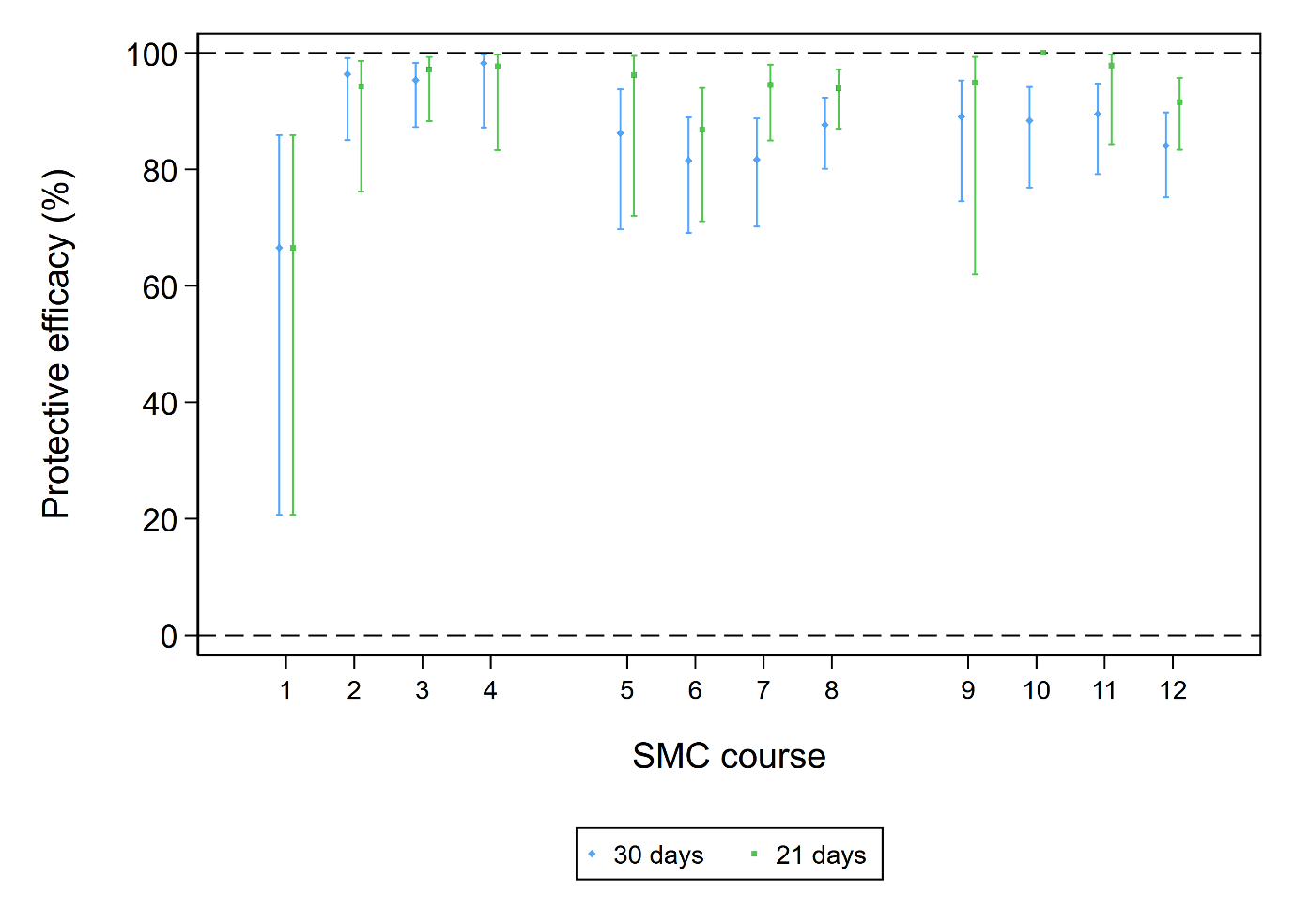


Footnote. Protective efficacy in 21- day and 30-day time strata after each SMC course, estimated from Cox regression models stratified on study country and with a robust standard error to account for multiple episodes. The hazard ratio comparing the Combined group to the RTS,S alone group was derived, and used to calculate protective efficacy as 1-HR, expressed as a percentage. There were no malaria cases in the Combined group in the first 21 days after SMC 10, so protective efficacy within 21 days is estimated as 100% (at it is not possible to estimate a CI).

**Figure S3**. Protective Efficacy by time since the final SMC cycle in each year. Data for SMC 4, SMC 8 and SMC 12 are shown separately.


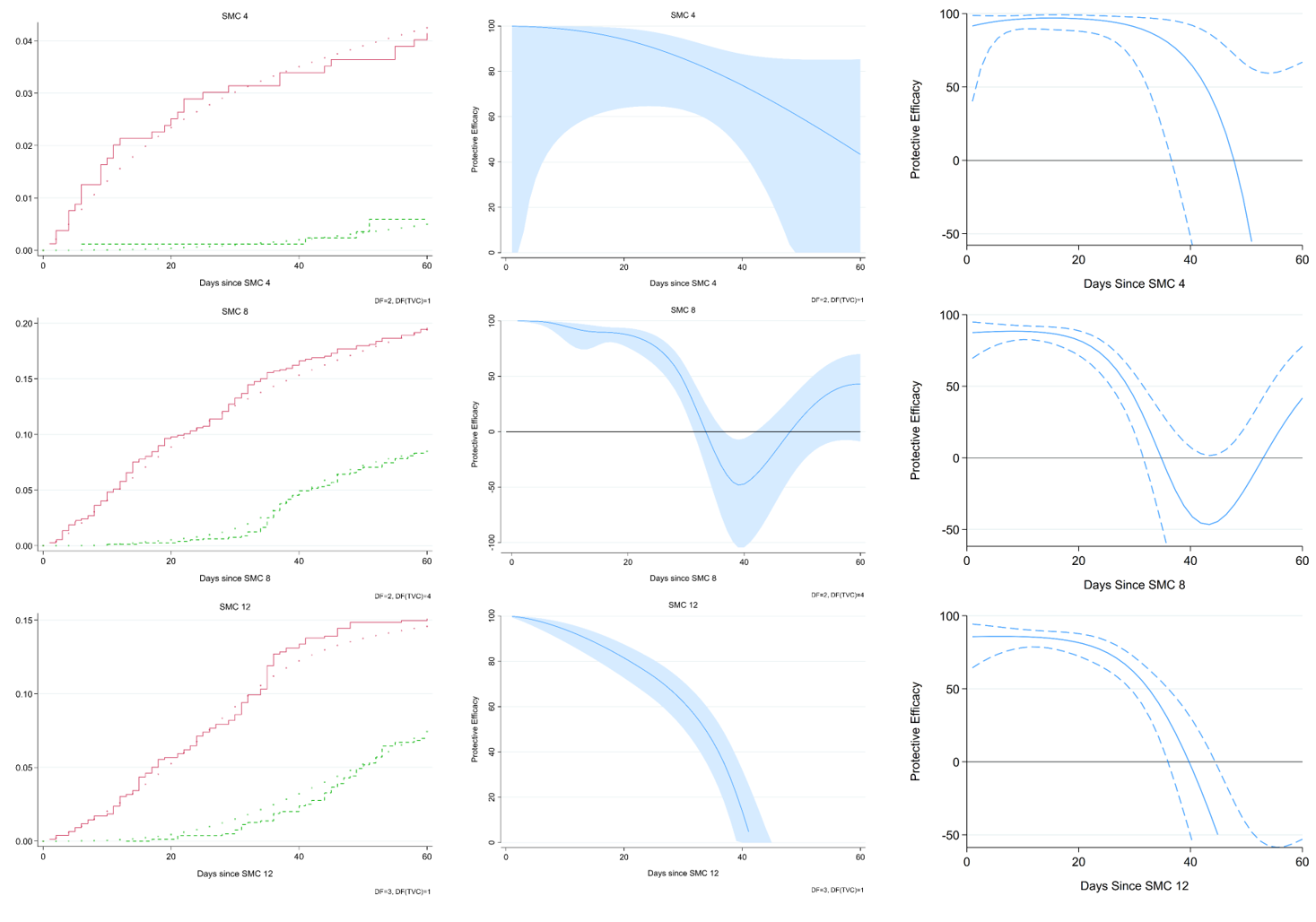


Footnote. The rows show results for SMC 4, SMC 8 and SMC 12, respectively (the final SMC cycle in each year of the study). Left hand panels show the observed cumulative hazard in the RTS,S alone and Combined intervention groups (solid lines), and the predicted cumulative hazard from the fitted flexible parametric survival model (dots). The middle panels show the estimated protective efficacy over time using the flexible parametric survival models (blue line) and the 95% CI (shaded area) for SMC 4, SMC 8 and SMC 12. For comparison, the estimated protective efficacy over time using the Smoothed Cox residuals is also shown for each cycle in the right hand panels.

**Figure** **S4**. Comparison of the SMC protective efficacy profile obtained in this study with the profile estimated for an earlier placebo-controlled trial of SMC.


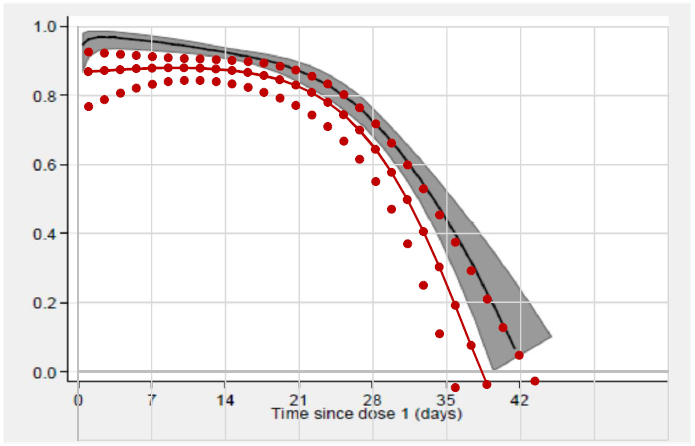


**Protective Efficacy**

Footnotes. Black and white image shows the estimated protective efficacy over time in a placebo-controlled trial of SMC among children with insecticide-treated nets (Konate et al. doi: 10.1371/journal.pmed.1000408; Dicko A et al. doi: 10.1371/journal.pmed.1000407 ; Milligan P. <https://researchonline.lshtm.ac.uk/id/eprint/4647454>.) The overlain plot (red dots, red line) show the estimated protective efficacy estimates in the present study, using Smoothed Schoenfeld residuals from Cox regression, as shown in Figure 6.
